# Aptamer-Modified Magnetic Beads in Biosensing

**DOI:** 10.3390/s18041041

**Published:** 2018-03-30

**Authors:** Harshvardhan Modh, Thomas Scheper, Johanna-Gabriela Walter

**Affiliations:** Institute of Technical Chemistry, Leibniz University of Hannover, Hannover 30167, Germany; modh@iftc.uni-hannover.de (H.M.); scheper@iftc.uni-hannover.de (T.S.)

**Keywords:** aptamer, magnetic beads, analytical applications, electrochemical assays, optical assays, point-of-care-testing

## Abstract

Magnetic beads (MBs) are versatile tools for the purification, detection, and quantitative analysis of analytes from complex matrices. The superparamagnetic property of magnetic beads qualifies them for various analytical applications. To provide specificity, MBs can be decorated with ligands like aptamers, antibodies and peptides. In this context, aptamers are emerging as particular promising ligands due to a number of advantages. Most importantly, the chemical synthesis of aptamers enables straightforward and controlled chemical modification with linker molecules and dyes. Moreover, aptamers facilitate novel sensing strategies based on their oligonucleotide nature that cannot be realized with conventional peptide-based ligands. Due to these benefits, the combination of aptamers and MBs was already used in various analytical applications which are summarized in this article.

## 1. Introduction

Aptamers are synthetic single-stranded (ss) DNA (deoxyribonucleic acid) or RNA (ribonucleic acid) molecules, which specifically bind to their target molecules with high affinity. They are selected by an iterative in vitro process termed systematic evolution of ligands by exponential enrichment (SELEX). In recent years, aptamers have been developed against a broad range of target molecules, including metal ions, small molecules, peptides, proteins, and even complex targets such as whole cells. Due to various advantages of aptamers, such as their animal free and cost effective production, high temperature stability, chemical stability, target versatility, and high affinity and selectivity for their targets, aptamers are appealing alternatives to antibodies (AB) for use in analytical applications [1].

**Figure 1:**
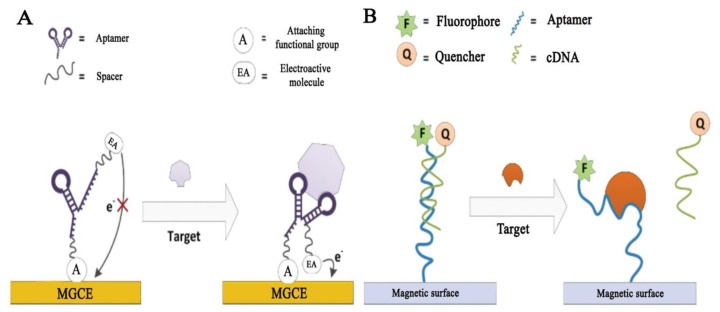
(**A**) Target-induced structure switching (TISS) type of assay. Here, the interaction between an aptamer and a target molecule leads to change in the conformation of the aptamer. The conformational changes can be exploited for signal generation, e.g., by using an electroactive molecule (EA) fused to the aptamer. In the figure, MGCE is a magnetic glass carbon electrode; (**B**) Target-induced dissociation (TID) type of assay. Here, the aptamer is hybridized with a complementary oligonucleotide (cDNA). The interaction between a target molecule and an aptamer leads to release of the cDNA sequence from the aptamer. The release of the cDNA can provide different types of signals in different assay formats, in the given example FRET-is used for signal generation. Adapted from [3] with permission. Copyright 2014, De Gruyter.

The specific advantages offered by aptamers include the easy modification with functional groups resulting in the possibility to control the orientation of the aptamer after immobilization. This controlled orientation facilitates high activity of immobilized aptamers, which is beneficial in their analytical applications [2]. In addition, aptamers can undergo considerable structural changes while interacting with target molecules. These changes have been extensively studied and exploited for the development of novel assays including target-induced structural switching (TISS) and target-induced dissociation (TID) of complementary oligonucleotides (Figure 1). These possibilities are specific for aptamers and allow for the design of sensing strategies even in cases, where conventional strategies, such as sandwich assays are not applicable. Moreover, aptamers can be regenerated and aptamer-modified sensors can be reused [3,4].

Recently, evolving analytical techniques and improved use of established methods have begun to incorporate micro- or nano-sized magnetic beads (MBs) [5]. The specific properties of MBs, such as colloidal stability of magnetic nanoparticles, homogenous size distribution, high and uniform magnetite content, a fast response to applied magnetic field, and presence of surface functional groups are essential for their analytical applications. The superparamagnetic properties of MBs permit the easy isolation of analytes from complex matrices, such as biological and environmental samples, by attaching specific ligands on the surface of MBs and separating the MBs-bound analytes with the aid of an external magnetic field. In order to provide different specificities, MBs can be functionalized with various reactive groups, such as amines, carboxyls, epoxyls, and tosyls, which can be used to immobilize high affinity ligands such as aptamers, proteins, antibodies, etc. according to required applications [6]. Suspended modified MBs with immobilized ligands can be highly recommended for the detection of analytes in complex matrices and the isolation of targets from larger volumes [7]. In some applications, the magnetic properties of the MBs, such as magnetic-relaxation switch, have been used for signal generation [8,9]. Lately, magnetic separation processes have been introduced in biotechnology for the purification of proteins [10,11,12,13,14,15,16], protein digestion [17,18,19], separation of cells [20,21], and analytical applications [18,19,22,23,24].

The combination of MBs with aptamers opens up new possibilities in a number of applications, such as sample preparation, wastewater treatment, water purification, disease therapy, disease diagnosis including magnetic resonance imaging, cell labelling and imaging, and biosensors. Within this review article, first a brief introduction of magnetic beads and their applications will be given in Section 2. In Section 3, the use of aptamer-modified MBs in biosensing will be described and recent examples will be highlighted.

## 2. Magnetic Beads and Their Biological Applications

Within this section magnetic beads will first be briefly introduced together with potential strategies to modify their surfaces. Consequently, the use of magnetic beads in some of the important biological applications such as separating biomolecules from complex matrices will be described.

### 2.1. Magnetic Beads and Their Modification

In 1792, William Fullarton described the separation of iron material with a magnet in a patent, which paved the way for applications of magnetic fields in separation techniques [6]. Initially, magnetic properties of sediments were used for separation. In 1852, a company from New York separated magnetite from apatite and later on, magnetite was separated from iron from brass fillings, turnings, metallic iron from furnace products and plain gauge etc. Gradually, magnetic separation evolved into complex and diverse commercial applications. In 1950, introduction of high gradient magnetic separation (HGMS) systems allowed faster and broad magnetic separation applications [25]. Towler et al. [26] reported the use of micron sized magnetite particles with adsorbent, manganese dioxide, on the surface to recover radium, lead and polonium from seawater samples. Remarkably, Safariková et al. introduced silanized magnetite particles (blue magnetite [27]) in magnetic solid-phase extraction (MSPE) for the first time to preconcentrate organic compounds [28] prior to analysis. Nowadays, magnetic separations have been widely used for protein purification [10,11,12,13,14,15,16], separation and purification of cells [20,21], and analytical applications [18,19,22,23,24].

**Figure 2:**
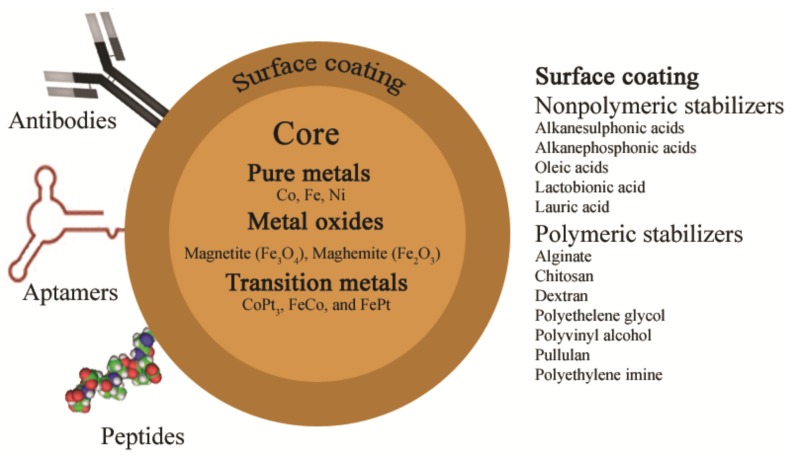
Composition of magnetic beads (MBs) used in analytical applications.

In recent applications, MBs are largely composed of a magnetic core, a surface coating, and specific binding ligands at the surface (Figure 2). Generally, magnetic cores can be composed from various materials, which exhibit magnetic properties. Largely, they consist of either pure metals (e.g., Co, Fe, and Ni) or their oxides. In addition, transition-metal-doped oxides and metal alloys, including CoPt_3_, FeCo, and FePt, are also good candidates. Among these magnetic materials, particularly iron oxides such as magnetite (Fe_3_O_4_) and maghemite (γ-Fe_2_O_3_) are considered to be the most attractive candidates for biological applications, owing to their strong magnetic property and biocompatibility [6]. U.S. Food and Drug Administration (FDA) and the European Medicines Agency (EMA) have approved the use of iron oxide MBs as magnetic resonance imaging (MRI) contrast agents [29]. Several approaches are available to synthesize the iron oxides. One widely used approach is co-precipitation from iron (III) chloride and iron (II) chloride solutions in the presence of aqueous ammonia solution [20,30,31]. Other methods are available including the extraction of bacterial magnetic particles (BMPs) from the flagella of magnetic bacteria from marine sediments [32,33].

Finely divided iron is highly reactive toward oxidizing agents in the presence of water or humid air. Thus, surface coating of magnetic particles is required to obtain physically and chemically stable systems. Such stabilization can be achieved by surface coating of the magnetic particles in numerous ways (Figure 2). Surface coating can be performed by using stabilising surface coating material, encapsulation into polymeric shells and into lipososmes [34].

Surface coating is primarily necessary to stabilize the newly formed surface of the particles and to prevent aggregation of the particles. Surface stabilization is generally achieved using nonpolymeric stabilizers based on organic monomers such as alkanesulphonic and alkanephosphonic acids, or phosphonates; oleic acid, lactobionic acid, lauric acid, or polymeric stabilizers, i.e., alginate, chitosan, dextran, polyethylene glycol, polyvinyl alcohol, pullulan, or polyethylene imine (Figure 2) [6]. Encapsulation into polymeric shells improves the water dispersibility and chemical and physical stability of the magnetic particles. In addition, the polymeric shell can provide a basis for conjugation of the magnetic particles to the targeting ligands by providing functional groups such as amine or carboxyl groups on the surface. Several ligands are available for the analytical purpose; the most popular among them are antibodies (ABs), aptamers, and peptides. The commonly used approach to attach ABs to MBs is the coupling of amine groups of the AB to carboxylated MBs via ethyl (dimethylaminopropyl) carbodiimide/*N*-hydroxysuccinimide (EDC/NHS) chemistry, but this can lead to random orientation of AB on the MBs. Different strategies have been attempted to avoid random orientation. For example, protein A can be used in order to immobilize the AB on the MB in a controlled orientation [35].

Currently, aptamers are also becoming popular for analytical applications. Several strategies are used for immobilising aptamers on the MBs. One of the popular strategies is to introduce amine groups on one terminus of the aptamer to allow immobilization to carboxylated MBs via EDC/NHS coupling. In contrast to AB immobilization, this results in highly oriented immobilization of aptamers, since only the terminal amine group can participate in coupling procedure. It is also possible to use streptavidin coated MBs and biotin-labelled aptamer for the attachment. Most important advantage here is the orientation of the aptamer, which can be easily controlled, as aptamers are chemically synthesized. During chemical synthesis, various modifications can be attached at defined positions within the aptamer sequence.

### 2.2. Separation of Biomolecules Using Modified MBs

The separation of biomolecules such as whole cells, proteins and peptides, and mRNA from complex matrices is very challenging. In this context, MBs modified with specific ligands allow to isolate the target molecule using strong magnets. This MB-based sample pre-treatment also allows to increase the concentration of target molecules in case of low concentration of the target molecules. In addition, time-consuming sample pre-treatment procedures like centrifugation, filtration and solid-phase extraction can be avoided [10]. In the following paragraphs, some exemplary applications of MBs to separate the biomolecules will be briefly discussed.

#### 2.2.1. Cell Separations

MB-based separation of cells from complex mixtures has become a popular tool and a valuable alternative to fluorescence activated cell sorting. In this process, specific aptamers or antibodies are anchored on the surface of magnetic particles. As example, Herr et al. immobilised an aptamer against leukemia cells on MBs. In this work, it was possible to specifically recognize the cells from complex mixtures including whole blood samples [36]. In a similar work by Zamay et al., MBs modified with aptamer against lung adenocarcinoma cells were used to separate circulating tumor cells (CTC) from human blood [37]. Interestingly, the applications of aptamer-modified MBs have been also integrated with microfluidic device to isolate cancer cell subpopulations [38]. Magnetic cell sorting (MACS) is used with antibodies since a long time [39]. The US FDA has also approved the Cellsearch^®^ system as in vitro diagnostic system for the detection of CTC in the clinic [40].

#### 2.2.2. mRNA Isolation

The isolation of mRNA using MBs is based on A-T pairing. Short sequence of dT (normally dT_(25)_) can be covalently attached to MBs, which will hybridise to dA-tail of mRNA and the isolation of mRNA can be possible within 15 min [41]. This technique eliminates the cumbersome steps used in traditional methods of mRNA isolation such as use of centrifugation and membrane-based spin columns. Importantly, MB-based isolation of mRNA fulfils the demand of automated systems and microfluidic devices, thereby promoting fast processing and high sample throughput [42]. The possibility of high throughput analysis has facilitated the identification of genetic aberrations in cancer cells [43], understanding of biochemical pathways [44] and phylogenetic studies [45].

#### 2.2.3. Protein and Peptide Enrichment

Traditionally, proteins are purified using expensive liquid chromatography systems, centrifuges, filters and other equipment. In addition, this purification process requires several steps and there is significant loss of protein/peptide at each step of purification. Purification using MBs reduces the amount of handling step and all the step can be done in a single test tube, which results in higher efficiency and reduced risk of contamination [46]. In some cases where intracellular proteins are targeted, it is even possible to combine the disruption of cells and separation of the protein form the complex mixture and thus shorten the total purification time. For example, Ni^2+^ or Co^2+^ coated MBs can be used to easily purify His-tagged proteins from cells [6]. Alternatively, aptamer-modified MBs can be used for the isolation of His-tagged proteins [47] or other proteins [48].

These methods can be used to isolate and enrich proteins prior to analysis. For example, immunomagnetic assays rely on MBs modified with antibodies (AB). Enzyme-linked immunosorbent assay (ELISA) is a gold-standard method for the detection of the protein in complex mixture. In immunomagnetic assays, the capturing antibody is immobilized on the MBs, which can reduce the incubation time and efficiency of the assay, as MBs remain suspended throughout the procedure [49]. Morozov et al. have developed an immunomagnetic assay for the detection of streptavidin in a micorofluidic device. This has been highly successful in reducing the assay time (three minutes) and better sensitivity (2 × 10^−17^ M) of the assay [50]. While Section 2 provided a brief overview on the general biological applications of magnetic beads, the next section will focus on the analytical applications of aptamer-modified magnetic beads.

## 3. Aptamer-Modified Magnetic Beads in Analytical Applications

In aptamer-modified MB-based assays, aptamers are used as binding ligands and MBs are mostly used for the separation of the analyte from complex matrices. MBs have also been coupled with antibodies, but aptamer-based assays offer prominent advantages such as high stability, broad dynamic range, prolonged shelf life, and low cross reactivity [51]. Moreover, aptamers are synthesized chemically, which facilitates straightforward and highly controlled modification with functional groups and different labels [52]. Moreover, the use of aptamers facilitates new assay designs that cannot be realized by using Abs. Aptamers can undergo significant conformational change upon binding to the target molecule. This can be exploited in target-induced structure switching (TISS)-based assays. Another assay format specific for aptamers is based on target-induced dissociation (TID) of complementary oligonucleotides. In case of TID, aptamers ability to hybridize with complementary sequences are used. Here, the helix structure formed between aptamer and its complementary sequence can be easily dissociated by competitive binding of the aptamer with its target (Figure 1). TISS as well as TID can be used for signal generation in various sensing strategies and are especially useful in cases were conventional formats such as sandwich assays are not applicable [3,53,54].

As shown in Table 1, a growing number of research groups are already using aptamer-modified MBs in analytical applications using various sensor designs. In the following sections, the applications have been broadly divided in the electrochemical, optical, piezoelectric and PCR-based assays.

### 3.1. Electrochemical

Since the first use of electrochemical (EC) assay for the detection of glucose by Clark and Lyons in 1963 [86], the applications have evolved in different kind of assays including ABs and aptamer-based assays. EC assays are generally rapid, highly sensitive, cost-effective and easy to miniaturize, which is highly attractive for the development of modern bioassays. In aptamer-based electrochemical assays, the change in electrochemical signals (current, voltage, and impedance) due to interaction of analytes and aptamers is measured [87].

EC assays can be broadly classified as amperometric, potentiometric, voltammetric, impedimetric, and electrogenerated chemiluminescence (ECL) assays, according to their working principles. In Table 1, recent work on aptamer-modified MBs in different electrochemical assays is summarized. 

#### 3.1.1. Voltammetric Assays

In voltammetric assays, a specific potential is applied to a working electrode in comparison to a reference electrode. The electrochemical reduction or oxidation at the surface of the working electrode results in the generation of a current. Here, amperometric assays are included as a subclass of voltammetric assays as, in amperometric assays also, a constant potential is applied on the working and reference electrode, and the change in current is measured over a period of time. In case of voltammetric assays, a potential range is applied and the changes in both, current and potential are observed. In both assays, the observed change in current is proportional to the concentration of the analyte. Different voltammetric modes are available such as cyclic voltammetry (CV), differential pulse voltammetry (DPV), squarewave voltammetry (SWV), and alternating current voltammetry (ACV) [88]. CV is majorly used to evaluate the electrode surface including purity, stability, and reproducibility of the electrode and to examine the aptamer immobilization on the electrode surface since it allows to check the redox behaviour over a wide potential range [58,88,89].

#### 3.1.2. Differential Pulse Voltammetry (DPV)

**Figure 3:**
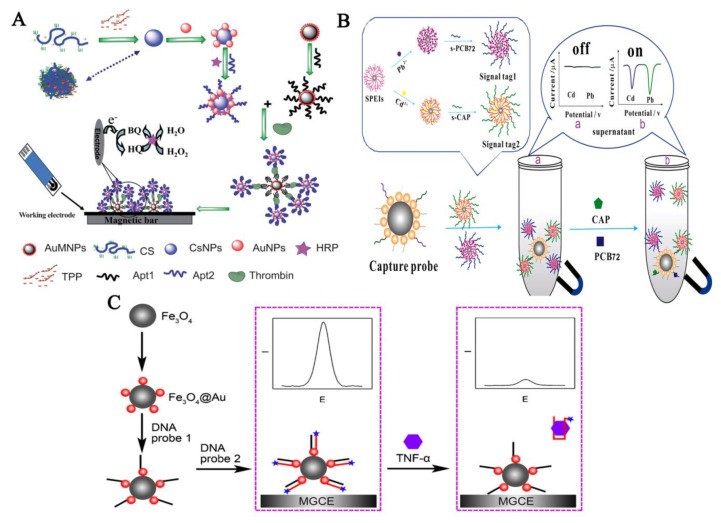
Utilization of MBs in aptamer-based electrochemical assays. (**A**) Using an electric signal mediator. Here, the electroactive molecules (HRP) were brought close to the electrode using aptamer-modified MBs. Reproduced with permission from [56]. Copyright 2012, Royal Society of Chemistry; (**B**) Signal-on type of electrochemical assay. The interactions between aptamers and the target molecules (Chloramphenicol and PCB 72) lead to generation of electrochemical signal. Reproduced with permission [90]. Copyright 2015, Elsevier; (**C**) Signal off type of electrochemical assay. In this type of assay, the interaction between the aptamer and the target molecule leads to reduction in electric signal. Reproduced with permission from [62]. Copyright 2017, Springer.

Due to its sensitivity and high selectivity, DPV is preferred in analytical applications. In DPV, different electroactive labels are used to generate the signal including small molecules and enzymes, such as horseradish peroxidase (HRP), alkaline phosphatise (AP), and glucose oxidase (GOD), etc. Among these enzyme labels, HRP is one of the most commonly used. For example, Zhao et al. [56] developed a highly sensitive and selective assay for the detection of thrombin (Figure 3A). In this assay, the capture probe was prepared to capture thrombin from the sample solution by immobilizing a thrombin aptamer-1 on MB-AuNP. The detection probe was prepared from another thrombin aptamer-2, horseradish peroxidase (HRP), thiolated chitosan (CS) nanoparticle and gold nanoparticle (CS-AuNP-HRP-Apt2). Presence of thrombin resulted in formation of the sandwich structure of MB-AuNP-Apt1/thrombin/Apt2-HRP-AuNP-CS. The sandwich structures were captured on the surface of a screen printed carbon electrode (SPCE) by a magnet located at the edge of SPCE. Due to the presence of HRP within the sandwich structure, the oxidation of hydroquinone (HQ) with H_2_O_2_ was dramatically accelerated. The observed electrochemical signal was proportional to the concentration of thrombin in the samples. A similar assay was also developed by Sun et al. [58] for the detection of human liver hepatocellular carcinoma cells (HepG2).

Centi et al. [57] have developed an electrochemical sandwich assay coupled to magnetic beads for the detection of thrombin in plasma. In this work, a microfluidic device was developed for the detection. The electrodes were screen-printed on a magnetic bar. Two different aptamers against thrombin were used where the first aptamer sequence was immobilised on MBs, to capture thrombin from the samples, using streptavidin-biotin interactions. The second aptamer was linked to alkaline phosphatase. The presence of thrombin in the sample resulted in current generation at the electrode, as more product was formed in the presence of alkaline phosphatase.

Zheng et al. [59] have developed an assay based on aptamer-conjugated to methylene blue for the detection of human platelet-derived growth factor BB (PDGF-BB). In this work, boronic acid modified SiO_2_@Fe_3_O_4_@PDA@AuNP composite was used to capture PDGF-BB from the sample. An aptamer against PDGF was linked to SiO_2_-methylene blue sphere, where methylene blue was used for its high electron-transfer efficiency. Due to the magnetic property of the electrode, the SiO_2_@Fe_3_O_4_@PDA@AuNP/PDGF/aptamer-SiO_2_-methylene blue could be easily enriched on the electrode surface. Later on, the electrochemical responses could be detected to quantify PDGF.

While in the previous examples, proteins were detected with aptamer-modified MBs, also small molecules are suitable targets, which can be detected e.g., by using the aptamer-specific TISS and TID-based assays. In this context, a TID-based label-free assay was developed by Yang et al. [60] for the detection of adenosine using thionine (Th) for generation of the electrochemical signal. In this work, adenosine aptamer was immobilised on MBs and an oligonucleotide complementary to the target-binding site of the aptamer (abbreviated as cDNA) was hybridized to the immobilized aptamer. Addition of adenosine resulted in the formation of aptamer-adenosine complex and the release of cDNA from the aptamer due to TID. The released cDNA was captured on the sensing electrode through DNA hybridization. As the cDNA is modified with thiol groups at the 5′ termini, AuNP can attach to the cDNA via the formation of S-Au bonding. Subsequently, the electroactive molecules, thionine, are adsorbed on the surfaces of the AuNPs and result in signal generation.

A TISS-based assay in which the structural changes of the aptamer were exploited for signal generation was developed by Wu et al. [61] for the detection of Hg^2+^ using streptavidin modified magnetic beads (Fe_3_O_4_-SA) and thionine, as electron mediator. In this work, Streptavidin-modified MBs (MB-SA) were immobilized onto the glassy carbon electrode (GCE) and provided magnetic character to the electrode. Then biotin-labelled aptamer against Hg^2+^ was immobilized to the electrode via SA-biotin interaction. Addition of Hg^2+^ resulted in a stable folded structure of thymine (T)-Hg^2+^-T where Th can easily intercalate. The detection of Hg^2+^ was achieved by recording the DPV signal of Th.

#### 3.1.3. Squarewave Voltammetry (SWV)

SWV is the popular pulse technique and widely considered for automatous and kinetic studies complementary to cyclic voltammetry. Here, TID-based assays are very popular and the interaction of the aptamer with the target molecule can result in electrical signal gain (signal-on) or signal suppression (signal-off).

Yan et al. [90] have developed an assay for the simultaneous detection of two different molecules, chloramphenicol and polychlorinated biphenyls-72 using a signal-on mechanism (Figure 3B). In this work, aptamers were immobilised on MBs and hybridised with a cDNA sequences attached to CdS or PbS QDs as electrochemical signal tracers. Binding of chloramphenicol caused the release of CdS QDs and binding of polychlorinated biphenyls-72 caused the release of PbS QDs. The released CdS and PbS QDs were simultaneously detected through the square wave voltammetry (SWV), which can switch the signals of the biosensor to “on’’ state. Hao et al. [63] have also developed an assay based on signal-on approach for the detection of ochratoxin A (OTA). In this work, addition of OTA caused the release of CdTe QDs modified with cDNA, from the MB-modified aptamers. After magnetic separation of aptamer-modified MBs, CdTe QDs remaining in the supernatant were dissolved by HNO_3_ and the concentration of Cd ions, which was directly proportional to the concentration of OTA, was detected by SWV.

Miao et al. [62] have developed a signal-off assay for the detection of TNF-α (Figure 3C). In this simple assay, methylene blue-tagged aptamer was immobilised on magnetic glassy carbon electrode (MGCE) using a cDNA. Addition of the target molecule resulted in release of methylene blue-tagged aptamer, resulting in decrease in electrochemical signal by SWV.

#### 3.1.4. Potentiometric Assays

In potentiometric sensors, the change in electric potential between two electrodes is detected by a field-effect transistor (FET) [91]. Here, the indicator electrode reports change in electric potential according to analyte concentration and the reference electrode provides constant electric potential.

Recently, Zhao et al. [66] have developed a potentiometric sensor for the detection of *Vibrio alginolyticus*, which is an opportunistic marine pathogen and can cause otitis, wound infection, and chronic diarrhoea in mammals. In this work, a cDNA sequences were immobilized on the surface of the magnetic beads using streptavidin-biotin interaction. The aptamer and H1/H2 (two different oligonucleotides) hybridize successively with the cDNA to form the DNA structure-modified magnetic beads. The resulted DNA structure can interact with protamine (polycation) due to electrostatic interactions, which can be detected by the polycation-sensitive electrode. When a sample containing *Vibrio alginolyticus* was added, the aptamers interact with the target due to its high affinity with the target. Consequently, the DNA structure disassembled and a reduction in potential was observed which was proportional the target concentration. A similar assay was also used for the detection of small molecules such as bisphenol A [92]. 

#### 3.1.5. Impedimetric Assays (EIS)

In impedimetric assays, electrochemical impedance spectroscopy (EIS) is popular due to its high sensitivity. EIS involves the analysis of the resistive and capacitive properties, which are based on the perturbation of a system at equilibrium by a small amplitude of excitation signal. EIS allows rapid and accurate detection of the small changes along the electrode by a transducer. The signal is enhanced by an amplifier, which makes EIS appealing in analytical applications. In addition, EIS is a simple technique and the detection in EIS does not require attachment of capture molecules to the electrodes and thus allows label-free detection.

Wang et al. [69] have developed a microfluidic analysis system assay for the detection of thrombin using aptamer-modified magnetic separation. In this work, thrombin aptamer-modified MBs were used to capture and separate the target protein from serum. Later on, the bound complex was injected into the microfluidic flow cell for impedance measurement. Similar analysis system was also developed by Jin et al. [68] for the detection of Cry1Ab protein to detect genetically modified crops. Another interesting impedimetric assay based on TID mechanism was developed by Lee et al. [93] for the detection of prostate-specific antigen (PSA). Combining PSA aptamer-modified magnetic nanoparticles with rolling circle amplification (RCA) has provided a better sensitivity of 0.74 pg mL^−1^ PSA in human serum.

#### 3.1.6. Electrogenerated Chemiluminescence

Electrogenerated chemiluminescence (ECL), also called electrochemiluminescence, refers to the emission of light via electron transfer reactions from electrochemically generated reagents. ECL combines the sensitivity and wide dynamic range from chemiluminescence (CL) with the advantages offered by electrochemical methods, such as simplicity, stability, and facility to be miniaturized [94,95]. Lately, ECL has been widely accepted in different analytical applications including fundamental studies to detecting trace amount of target molecules. Among different luminophores, use of luminol, quantum dots (QDs) and ruthenium(II) complexes, have been widely employed in ECL assays [96].

Ke et al. [70] have developed an assay for the detection of β-amyloid (Aβ) using a sandwich-type ECL sensing platform. In this work, Ru(bpy)_3_^2+^ was used as ECL donor and gold nanorods (GNRs) were used as ECL acceptor. Here, resonance energy transfer (RET) donor nanohybrids were prepared with mesoporous carbon nanospheres (MCNs)@nafion/Ru(bpy)_3_^2+^/Aβ antibody. After incubation with target Aβ protein and GNRs-attached aptamer, prominent decrease in ECL signal was observed due to the quenching effect between Ru(bpy)_3_^2+^ and GNRs. This innovative approach performed well with sensitivity of 4.2 fg mL^−1^ in real Alzheimer’s patient cerebrospinal fluid samples.

In a TID-based approach, Wang et al. [71] have developed a novel ECL sensing system for the detection of HL-60 cancer cells. Here, Ag-polyamidoamine (PAMAM) was prepared and functionalized with cDNA and bio-bar-code DNA (bbcDNA). The prepared composite was hybridized with the aptamer-modified MBs. Addition of HL-60 cancer cells resulted in the release of cDNA-Ag-PAMAM composite in the supernatant. For the detection, an oligonucleotide complementary to cDNA was immobilised on the electrode surface resulting in hybridization of released cDNA-Ag-PAMAM.

### 3.2. Optical

Optical assays have been widely used due to their specific advantages such as high sensitivity, quick response, high signal-to-noise ratio, reduced cost of manufacture, and relatively simple operation. Aptamers are preferred ligands in optical assays due to flexibility in modification with various fluorophores and other labels. As already described for electrochemical assays, application of aptamer-modified MBs can be highly advantageous due to easy separation of the target molecule from the complex matrices, which offers high signal-to-noise ratio during measurement. The assays can be classified based on the detection principle including fluorescence, colorimetry, chemiluminescence, surface plasmon resonance (SPR), and Raman scattering.

#### 3.2.1. Fluorescence Based Assays

Aptamer-based fluorescence assays can be mainly divided into labelled and label-free assays. Easy modification of aptamers with fluorophores and quenchers during chemical synthesis facilitates the design of various assays. 

**Figure 4:**
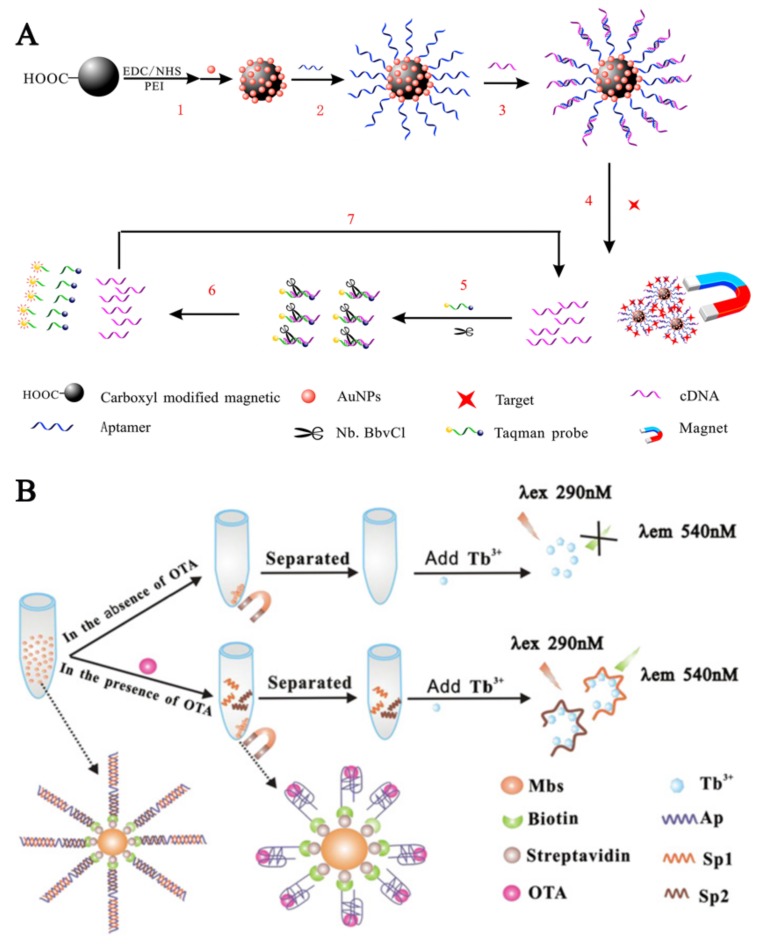
Fluorescence-based assays. (**A**) Combining fluorophores and quencher molecules. Here the interaction between the aptamer and the target molecule leads to the release of quencher molecule and the increase of fluorescence signal. Reproduced with permission from [97]. Copyright 2017, Elsevier; (**B**) Label-free assay. Being oligonucleotides, aptamers can specifically interact with dyes specific for ssDNA or dsDNA. In this example, Tb^3+^ was used which interacts specifically with ssDNA (cDNA), which was released due to TID from ochratoxin A (OTA) aptamer. Reproduced with permission from [72]. Copyright 2013, Elsevier.

Luo et al. [97] developed a nicking enzyme assisted signal amplification (NEASA)-based assay relying on TID mechanism (Figure 4A). In their work, ampicillin aptamer was immobilised on MBs and attached to a complementary sequence (cDNA) through Watson-Crick base pairing. Addition of ampicillin resulted in release of cDNA which can bind to Taqman probe having fluorescent and quencher probes at opposite end. The nicking enzyme cleaved the Taqman probe only when it was bound to cDNA. The decrease in fluorescence signal was proportional to the concentration of ampicillin.

Upconversion nanoparticles (UCNPs), nanocrystals containing lanthanide ions, emerged as an important fluorophor, as they lack autofluorescence and their use result in high signal-to-noise ratio. In addition, the optical properties of UCNP can be tuned with different lanthanide dopants such as Er^3+^, Tm^3+^, and Ho^3+^ [98,99]. Fang et al. [73] developed an assay for the detection of circulating tumor cells (CTC) using UCNPs. In their work, UCNPs were modified with the aptamer and biotinylated-PEG. Aptamer was used to recognize CTC and biotinylated-PEG was used to attach UCNP to the MBs. Here, whole blood samples were mixed with the modified UCNPs and it was possible to detect as low as 10 cells into 0.5 mL of whole blood samples. Efforts have been also made to further increase the sensitivity of fluorescence-based assays. In this context, Wang et al. [100] used RuBpy-doped silica nanoparticles (RSiNPs), which are highly photostable and provide significant enhancement in fluorescent signal when compared with single RuBpy dye molecules. In this work, aptamer-modified MBs were used to separate thrombin from human serum.

Aptamer-modified MBs have been also used in enzyme-linked aptamer sandwich assays. John Bruno et al. [101] used aptamer-modified MBs to capture *Campylobacter jejuni* from the samples. Later on, a second aptamer modified with QDs was introduced. The bound complex was brought on the photo detector using external magnet. The whole detection procedure could be finished in 15 min and resulted in high sensitivity. Similar detection principle was also used by Hao et al. for the detection of thrombin [102].

To reduce the labelling cost and reducing the effect of labelling on aptamer conformation, label free assays have attracted big attention. Being DNA sequences, aptamers can also bind to DNA binding chemicals, such as crystal violet [103], SYBR Green I (SGI) [104,105], 4′,6-diamidino-2-phenylindol (DAPI) [106], malachite green [107], OliGreen [108] and terbium (III) (Tb^3+^). Zhang et al. [72] developed a label-free fluorescent aptasensor based on the Tb^3+^, structure-switching of anti-OTA aptamer and MBs for the detection of ochratoxin A in wheat (Figure 4B).

#### 3.2.2. Colorimetric Assay

Colorimetric assays are widely used in analytical application due to simplicity of the measurement. Among aptamer-based colorimetric assays, AuNP-based assays are widely used. MBs-based separation in these assays provide better signal-to-noise ratio and sensitivity.

For example, Liang et al. [109] reported an aptamer-protein interaction-induced aggregation assay for the detection of thrombin. In their work, two different aptamers against human α-thrombin were immobilised on nanoroses (MB-AuNP core-shell structure in a flowerlike shape). Addition of human α-thrombin in solution resulted in aggregation of nanoroses and thus in a characteristic change in UV-Vis absorption spectra of the colloid. An interesting assay was also developed by Wang et al. [75] for the detection of Hg(II) based on hybridization chain reaction (HCR). In this work, aptamer against Hg(II) were immobilized on the MB-AuNP. HCR process is inhibited in the presence of Hg(II) enabling less methylene blue to intercalate into the dsDNA structure.

The use of peroxidase-like activity of magnetic nanoparticles is exploited in different colorimetric assays. Kim et al. [110] reported an assay for the detection of metal ions. Here, the aptamers were adsorbed on the positively charged surface of MBs, which reduces the catalytic activity of MBs. Addition of the target molecules released the adsorbed aptamers from MBs surface and MBs recover the peroxidase-like activity. Similar assays were also developed for the detection of ochratoxin A in cereal samples [111] and thrombin in blood plasma [112].

#### 3.2.3. Chemiluminescence Assays

In chemiluminescence (CL) assays, the light emitted during a chemical reaction is detected. The unique characteristics of CL include high sensitivity, wide dynamic ranges and simple instrumentation. In case of CL, an excitation light-source is not required which is highly cost effective.

A label-free CL detection of adenosine in human serum was realized by Yan et al. [113]. In their work, 3,4,5-trimethoxyl-phenylglyoxal (TMPG) was used as the signalling molecule for CL, as TMPG can intercalate with guanine (G) nucleobases. Firstly, the cDNA was immobilized on the surface of MBs and hybridized with a G-rich adenosine aptamer. Addition of adenosine containing sample caused the release of the aptamer from MBs modified with cDNA and a decrease in CL signal was observed, which was proportional to adenosine concentration.

**Figure 5:**
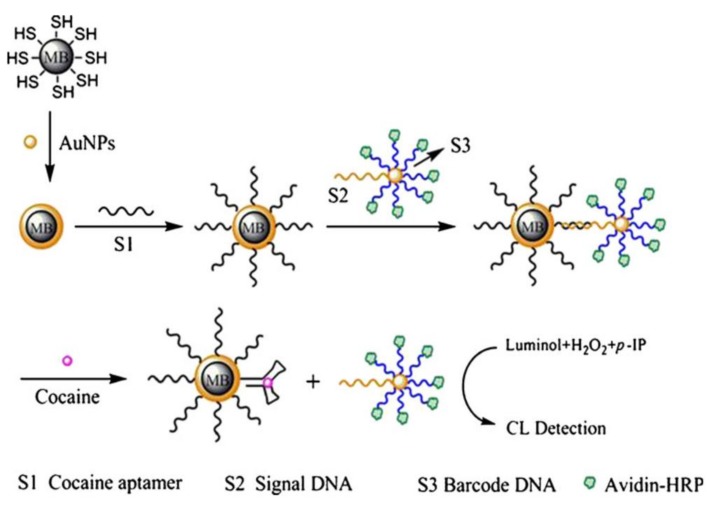
Chemiluminescence assay. This assay is based on TID. Here, the interaction between the aptamer and the target molecule (cocaine) caused the release of cDNA attached to HRP-modified AuNPs. Released HRP generated chemiluminescence signal which was proportional to cocaine concentration. Reproduced with permission from [114]. Copyright 2011, Springer.

In contrast, HRP-based catalysis can be widely used for the generation of CL signal. As an example for CL assays using HRP, Li et al. [114] immobilized aptamers directed against cocaine on the surface of AuNP-functionalized MBs (MB-AuNP). Therefore, aptamers were hybridized with the cDNA immobilized on the double-functional AuNP modified with HRP (HRP-AuNP). When cocaine was introduced, a dissociation of the aptamer was achieved due to binding of the aptamer to cocaine. Consequently, HRP-AuNP were eluted from the MB-AuNP due to target-induced dissociation (TID). The recorded CL signals were proportional to the concentration of cocaine (Figure 5).

#### 3.2.4. Surface-Enhanced Raman Scattering-Based Assays

Surface-enhanced Raman scattering (SERS) relies on the principle that the Raman scattering intensity of molecules will be greatly improved after their adsorbtion onto the metal surface. 

**Figure 6:**
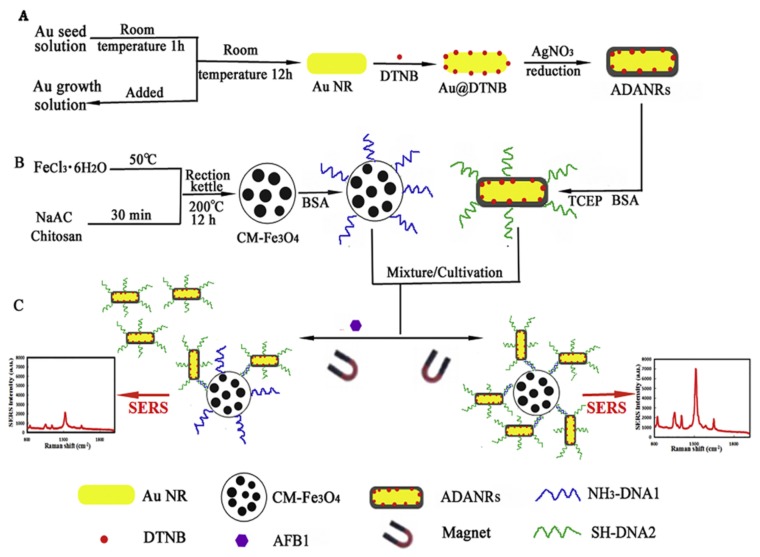
Surface-enhanced Raman scattering-based assays. (**A**) Immobilization of aflatoxin B1 (AFB1) aptamer on gold nanorods (AuNRs). (**B**) Immobilization of cDNA on chitosan-modified MBs. (**C**) Schematic representation of AFB1 measurement. Here, the binding of AFB1 induced the release of cDNA and, in turn, AuNRs from the MBs and a decrease in SERS signal was observed. Reproduced with permission from [78]. Copyright 2018, Elsevier.

Quansheng et al. [78] developed a SERS assay for ultrasensitive detection of aflatoxin B1 (AFB1) detection in peanut oil (Figure 6). In this study, AFB1 aptamers were immobilised on the MBs and cDNA was immobilised on gold nanorods (cDNA-AuNRs). Presence of AFB1 resulted in the release of cDNA-AuNRs and decrease in SERS signal was observed. In an interesting work, aptamer-conjugated magnetic beads were used for the separation of circulating tumor cells from whole blood samples and the tumor cells were detected using surface-enhanced Raman scattering imaging [115].

Yoon et al. [116] developed SERS-based magnetic aptasensors for the detection of thrombin in serum samples. In this work, two different aptamers against thrombin were used for the detection. One aptamer was immobilised on MBs and second aptamer was immobilised on AuNP-coated with Raman reporter molecules, X-rhodamine-5-(and -6)-isothiocyanate (XRITC). Addition of thrombin resulted in the formation of sandwich aptamer complexes and an increase of SERS signal according to thrombin concentration in the sample was observed.

### 3.3. Piezoelectric Assays

Since the discovery of piezoelectric effect in 1880 by the Curie brothers, the piezoelectric effect has been very popular in analytical applications. Lately, the progress made in the fields of microelectronics and microfluidics further promotes the development of label-free piezoelectric assays [117]. Particularly, quartz-crystal microbalance (QCM)-based assays have become popular in analytical applications of aptamers. Utilizing MBs in these assays is highly advantageous due to their inherent piezoelectric properties, and potential to concentrate the analyte molecules at the QCM surface [118].

Ozalp et al. [81] developed a QCM biosensor for the detection of *Salmonella* cells in food samples. Here, aptamer-modified MBs were, firstly, used to capture the target pathogens from the food samples. The magnetically separated pathogens were detected by QCM sensor and 100 cfu mL^−1^ *Salmonella* cells could be detected in milk samples. In a similar assay, Pan et al. [82] detected leukemia cells in complex matrices. In this work, aptamer-modified MBs were used to capture leukemia cells from the biological sample and to approximate them to the QCM sensor using an external magnet.

An interesting approach was used by Song et al. [119] for the detection of ATP via DNAzyme-activated and aptamer-based target-triggered circular amplification. In this work, AuNPs were used for mass amplification and captured on the modified gold electrode. The amplification scheme involved circular nucleic acid strand-displacement polymerization, aptamer binding strategy and DNAzyme signal amplification. Presence of ATP resulted in a two-cycle amplification process, triggered by the aptamer recognition of a target molecule.

### 3.4. PCR-Based Assays

Being oligonucleotide sequences, aptamers can be easily amplified and quantified using qPCR with high sensitivity and reproducibility. Recently, different assays based on this property of aptamers have been developed including Apta-qPCR, micromagnetic aptamer PCR (MAP), and assays involving PCR-based amplification strategies as loop-mediated isothermal amplification (LAMP), rolling circle amplification, and isothermal signal amplification, proximity ligation assays, and nuclease protection assays. In these assays, MBs provides opportunity to separate target-bound and unbound aptamers, which is very important to get minimum background and high signal-to-noise ratios and in turn high sensitivity and reproducibility.

**Figure 7:**
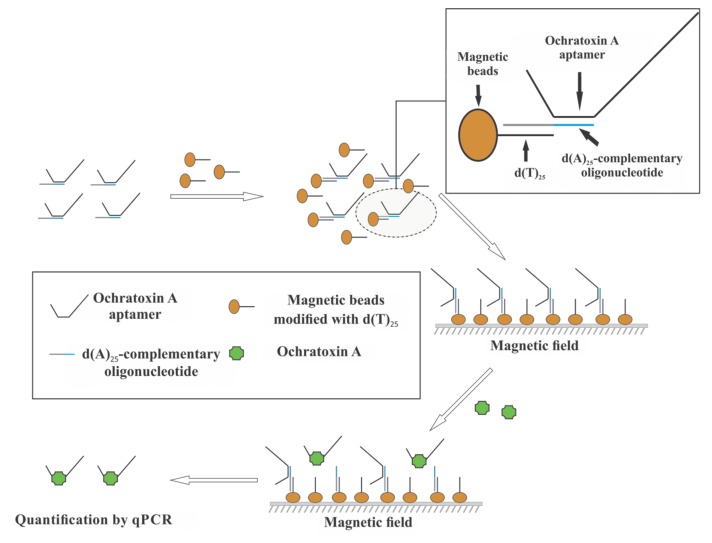
Apta-qPCR. This assay is based on TID, where the interaction of the target molecules (ochratoxin A) caused the release of aptamer from the cDNA-modified MBs. The released aptamers were quantified using qPCR. Reproduced with permission from [53]. Copyright 2017, Wiley.

Our group [53] developed an Apta-qPCR assay for the detection of ochratoxin A in beer samples (Figure 7). In this work, ochratoxin A aptamer was hybridized to a corresponding cDNA, which was immobilized on MBs. Addition of the target molecules caused TID of aptamer from the MBs and the released aptamers were quantified using qPCR. This assay was able to detect 0.009 ng mL^−1^ OTA in beer samples. Similar assay was also used for the quantification of ATP present in HeLa cell lysate with the sensitivity of 17 nM ATP [54].

Csordas et al. [84] developed an interesting concept for the detection of PDGF-BB using combination of antibody and aptamer in MAP. In this work, high-gradient magnetic field sample preparation was integrated within a microfluidic device with aptamer-based real-time PCR readout. Antibody-modified MBs were used for capturing PDGF-BB and an aptamer against PDGF-BB, which was modified with flanking PCR primer sequences, was added after washing. The bound aptamers were quantified using qPCR. 

Ozalp et al. [85] developed a qPCR-based assay where aptamer-modified MBs were used to preconcentrate the *Escherichia coli* or *Salmonella typhimurium*. Later on, bacterial genomes were extracted which was quantified using qPCR. A similar assay was also developed by Feng et al. [120] for the detection of *Listeria monocytogenes* using a LAMP assay.

## 4. Conclusions and Future Trends

Aptamers are attractive bioreceptors in analytical applications due to their small size, animal free- and cost effective production, high stability (especially DNA aptamers), target versatility, high binding affinity and selectivity for their target molecules. In addition, several properties of aptamers including ease of chemical modification, measurable structural changes induced upon interaction of the aptamer with the target molecule, and the potential to amplify aptamers via PCR are advantageous in comparison to other binding ligands such as ABs. Due to these significant advantages of aptamers, they have been widely used for the detection of different analytes ranging from metal ions, small molecules, proteins to whole cells in diverse assay formats. In this review article, the focus was put on the magnetic bead-based analytical applications of aptamers. Utilization of MBs in aptamer-based applications allows to rapidly detect the analyte in the complex matrices with high signal-to-noise ratio. Recent developments in the synthesis of MBs resulted in MBs with better homogenous size distribution, high and uniform magnetite content, and a fast response to applied magnetic field, as well as high colloidal stability of magnetic nanoparticles.

In this review, different assay formats have been discussed where MBs were coupled with aptamers for the analytical applications. In many of the applications, MBs help to separate the target molecule from complex matrices. In some applications the aptamer-modified beads are also enriched directly on the sensor surface, thereby representing a surface modification used to immobilize the target. In few applications, magnetic properties of the MBs, such as magnetic-relaxation switch, have been used for signal generation.

Application of aptamer in analytical techniques is still in development phase, as many commercial applications use ABs. Slowly but steadily, aptamers are developed against a range of molecules. For some of them the development of other ligands, such as antibodies, is not easy, e.g., due to low immunogenicity or high toxicity. Moreover, aptamers seem to be especially advantageous for the detection of small molecules. In this context, TISS and TID mechanism provide the possibility to design assays that can detect small molecules, while other strategies like sandwich assays are not suitable for detection of small molecules. These advantages of aptamers and their combination with those of MBs, can further boost the development of new analytical procedures. The use of MBs in these assays can result in rapid detection of the target molecules even within complex matrices with no need for time-consuming sample pre-treatment procedures. Taking together the strengths of aptamers and MBs can therefore be especially advantageous in the development of POCT, where complex samples have to be analyzed within minutes.

## Figures and Tables

**Table 1:** Examples of coupling magnetic beads in aptamer-based analytical applications.

Method	Analytes	Detection Limit	Reference
**Electrochemical**			
**Voltammetric**			
Differential pulse voltammetry (DPV)	Human activated protein C	2.35 µg mL^−^^1^	[55]
DPV	Thrombin	5.5 fM	[56]
DPV	Thrombin	5 nM	[57]
DPV	Human liver hepatocellular carcinoma cells (HepG2)	15 cells mL^−^^1^	[58]
DPV	Platelet derived growth factor BB (PDGF BB)	0.22 fM	[59]
DPV	Adenosine	0.05 nM	[60]
DPV	Hg^2+^	0.33 nM	[61]
Squarewave voltammetry (SWV)	Tumor necrosis factor-alpha (TNF-α)	10 pg mL^−^^1^	[62]
SWV	Ochratoxin A	0.07 pg mL^−^^1^	[63]
**Potentiometric**			
Potentiometric carbon-nanotube aptasensor	Variable surface glycoprotein from African Trypanosomes	10 pM	[64]
Direct Potential Measurement	*Listeria monocytogenes*	10 cfu mL^−^^1^	[65]
Chronopotentiometry	*Vibrio alginolyticus*	10 cfu mL^−^^1^	[66]
**Impedimetric**			
Electrochemical impedance spectroscopy	*Salmonella*	25 cfu mL^−^^1^	[67]
Impedimetric microfluidic analysis	Protein Cry1Ab	0.015 nM	[68]
Microfluidic impedance device	Thrombin	0.01 nM	[69]
**Electrogenerated Chemiluminescence**			
Electrochemiluminescence resonance energy transfer system	β-amyloid	4.2 × 10^−6^ ng mL^−^^1^	[70]
Ratiometric electrochemiluminescence	Cancer cells	150 cells mL^−^^1^	[71]
**Optical**			
**Fluorescence**			
Signal-on fluorescent aptasensor	Ochratoxin A	20 pg mL^−^^1^	[72]
Aptamer-conjugated upconversion nanoprobes assisted by magnetic separation	Circulating tumour cells	20 cells mL^−^^1^	[73]
Enzyme-linked aptamer assay	Oxytetracycline	0.88 ng mL^−^^1^	[74]
**Colorimetric**			
Colorimetric assay (Methylene Blue-based)	Hg(II)	0.7 nM	[75]
**Chemiluminescence**			
Chemiluminescent	Hepatitis B Virus	0.1 ng mL^−^^1^	[76]
Chemiluminescence (integrated microfluidic system)	Glycated haemoglobin	0.65 g dL^−^^1^ for HbA1c and 8.8 g dL^−^^1^ for Hb	[77]
**Surface enhanced Raman scattering**			
Molecular embedded SERS aptasensor	Aflatoxin B1	0.0036 ng mL^−^^1^	[78]
Universal SERS aptasensor	Aflatoxin B1	0.54 pg mL^−^^1^	[79]
Induced Target-Bridged Strategy	platelet derived growth factor BB	3.2 pg mL^−^^1^	[80]
**Piezoelectric**			
Quartz crystal microbalance sensor	*Salmonella enterica*	100 cfu mL^−1^	[81]
Magnet-quartz crystal microbalance system	Acute leukemia cells	8 × 10^3^ cells mL^−1^	[82]
**PCR-based assays**			
Apta-qPCR	ATP	17 nM	[54]
Apta-qPCR	Ochratoxin A	0.009 ng mL^−^^1^	[53]
Rolling circle amplification	Cocaine	0.48 nM	[83]
Micromagnetic aptamer PCR	PDGF-BB	62 fM	[84]
Real-time PCR	*Escherichia coli*	100 cfu mL^−1^	[85]
**Magnetic relaxation**			
Magnetic nanosensors	CCRF-CEM cell	40 cells mL^−^^1^	[8]
Magnetic relaxation switch	*Pseudomonas aeruginosa*	50 cfu mL^−1^	[9]

## References

[1] Sun H., Zu Y. (2015). A highlight of recent advances in aptamer technology and its application. Molecules.

[2] Witt M., Walter J.-G., Stahl F. (2015). Aptamer microarrays—Current status and future prospects. Microarrays.

[3] Walter J.-G., Heilkenbrinker A., Austerjost J., Timur S., Stahl F., Scheper T. (2012). Aptasensors for small molecule detection. Z. Naturforsch. B.

[4] Urmann K., Walter J.-G., Scheper T., Segal E. (2015). Label-free optical biosensors based on aptamer-functionalized porous silicon scaffolds. Anal. Chem..

[5] Rocha-Santos T.A.P. (2014). Sensors and biosensors based on magnetic nanoparticles. TRAC Trend Anal. Chem..

[6] Aguilar-Arteaga K., Rodriguez J.A., Barrado E. (2010). Magnetic solids in analytical chemistry: A review. Anal. Chim. Acta.

[7] Kudr J., Klejdus B., Adam V., Zitka O. (2017). Magnetic solids in electrochemical analysis. TRAC Trend Anal. Chem..

[8] Bamrungsap S., Chen T., Shukoor M.I., Chen Z., Sefah K., Chen Y., Tan W. (2012). Pattern recognition of cancer cells using aptamer-conjugated magnetic nanoparticles. ACS Nano.

[9] Jia F., Xu L., Yan W., Wu W., Yu Q., Tian X., Dai R., Li X. (2017). A magnetic relaxation switch aptasensor for the rapid detection of Pseudomonas aeruginosa using superparamagnetic nanoparticles. Microchim. Acta.

[10] Khng H.P., Cunliffe D., Davies S., Turner N.A., Vulfson E.N. (1998). The synthesis of sub-micron magnetic particles and their use for preparative purification of proteins. Biotechnol. Bioeng..

[11] Liao M.-H., Chen D.-H. (2002). Fast and efficient adsorption/desorption of protein by a novel magnetic nano-adsorbent. Biotechnol. Lett..

[12] Bucak S., Jones D.A., Laibinis P.E., Hatton T.A. (2003). Protein separations using colloidal magnetic nanoparticles. Biotechnol. Prog..

[13] Shao D., Xu K., Song X., Hu J., Yang W., Wang C. (2009). Effective adsorption and separation of lysozyme with PAA-modified Fe_3_O_4_@silica core/shell microspheres. J. Colloid Interface Sci..

[14] Oktem H.A., Bayramoglu G., Ozalp V.C., Arica M.Y. (2007). Single-step purification of recombinant thermus aquaticus DNA polymerase using DNA-aptamer immobilized novel affinity magnetic beads. Biotechnol. Prog..

[15] Shukoor M.I., Natalio F., Tahir M.N., Ksenofontov V., Therese H.A., Theato P., Schröder H.C., Müller W.E., Tremel W. (2007). Superparamagnetic γ-Fe_2_O_3_ nanoparticles with tailored functionality for protein separation. Chem. Commun..

[16] Sun Y., Ding X., Zheng Z., Cheng X., Hu X., Peng Y. (2007). A novel approach to magnetic nanoadsorbents with high binding capacity for bovine serum albumin. Macromol. Rapid Commun..

[17] Jeng J., Lin M.F., Cheng F.Y., Yeh C.S., Shiea J. (2007). Using high-concentration trypsin-immobilized magnetic nanoparticles for rapid in situ protein digestion at elevated temperature. Rapid Commun. Mass Spectrom..

[18] Li Y., Xu X., Deng C., Yang P., Zhang X. (2007). Immobilization of trypsin on superparamagnetic nanoparticles for rapid and effective proteolysis. J. Proteome Res..

[19] Lin S., Yao G., Qi D., Li Y., Deng C., Yang P., Zhang X. (2008). Fast and efficient proteolysis by microwave-assisted protein digestion using trypsin-immobilized magnetic silica microspheres. Anal. Chem..

[20] Chen W., Shen H., Li X., Jia N., Xu J. (2006). Synthesis of immunomagnetic nanoparticles and their application in the separation and purification of CD34^+^ hematopoietic stem cells. Appl. Surf. Sci..

[21] Antoine J.-C., Rodrigot M., Avrameas S. (1978). Lymphoid cell fractionation on magnetic polyacrylamide-agarose beads. Immunochemistry.

[22] Krogh T.N., Berg T., Højrup P. (1999). Protein analysis using enzymes immobilized to paramagnetic beads. Anal. Biochem..

[23] Gatto-Menking D.L., Yu H., Bruno J.G., Goode M.T., Miller M., Zulich A.W. (1995). Sensitive detection of biotoxoids and bacterial spores using an immunomagnetic electrocheminescence sensor. Biosens. Bioelectron..

[24] Guesdon J.-L., Avrameas S. (1977). Magnetic solid phase enzyme-immunoassay. Immunochemistry.

[25] Yavuz C.T., Prakash A., Mayo J., Colvin V.L. (2009). Magnetic separations: From steel plants to biotechnology. Chem. Eng. Sci..

[26] Towler P.H., Smith J.D., Dixon D.R. (1996). Magnetic recovery of radium, lead and polonium from seawater samples after preconcentration on a magnetic adsorbent of manganese dioxide coated magnetite. Anal. Chim. Acta.

[27] Šafařík I., Šafaříková M., Vrchotová N. (1995). Study of sorption of triphenylmethane dyes on a magnetic carrier bearing an immobilized copper phthalocyanine dye. Collect. Czech. Chem. Commun..

[28] Šafaříková M., Šafařík I. (1999). Magnetic solid-phase extraction. J. Magn. Magn. Mater..

[29] Hsing I., Xu Y., Zhao W. (2007). Micro-and nano-magnetic particles for applications in biosensing. Electroanalysis.

[30] Quy D.V., Hieu N.M., Tra P.T., Nam N.H., Hai N.H., Thai Son N., Nghia P.T., Anh N.T.V., Hong T.T., Luong N.H. (2013). Synthesis of silica-coated magnetic nanoparticles and application in the detection of pathogenic viruses. J. Nanomater..

[31] Chen C.-T., Chen Y.-C. (2005). Fe_3_O_4_/TiO_2_ core/shell nanoparticles as affinity probes for the analysis of phosphopeptides using TiO_2_ surface-assisted laser desorption/ionization mass spectrometry. Anal. Chem..

[32] Blakemore R. (1975). Magnetotactic bacteria. Science.

[33] Matsunaga T., Maeda Y., Yoshino T., Takeyama H., Takahashi M., Ginya H., Aasahina J., Tajima H. (2007). Fully automated immunoassay for detection of prostate-specific antigen using nano-magnetic beads and micro-polystyrene bead composites, ‘Beads on Beads’. Anal. Chim. Acta.

[34] Canfarotta F., Piletsky S.A. (2014). Engineered magnetic nanoparticles for biomedical applications. Adv. Healthc. Mater..

[35] Paleček E., Fojta M. (2007). Magnetic beads as versatile tools for electrochemical DNA and protein biosensing. Talanta.

[36] Herr J.K., Smith J.E., Medley C.D., Shangguan D., Tan W. (2006). Aptamer-conjugated nanoparticles for selective collection and detection of cancer cells. Anal. Chem..

[37] Zamay G.S., Kolovskaya O.S., Zamay T.N., Glazyrin Y.E., Krat A.V., Zubkova O., Spivak E., Wehbe M., Gargaun A., Muharemagic D. (2015). Aptamers selected to postoperative lung adenocarcinoma detect circulating tumor cells in human blood. Mol. Ther..

[38] Labib M., Green B., Mohamadi R.M., Mepham A., Ahmed S.U., Mahmoudian L., Chang I.-H., Sargent E.H., Kelley S.O. (2016). Aptamer and antisense-mediated two-dimensional isolation of specific cancer cell subpopulations. J. Am. Chem. Soc..

[39] Miltenyi S., Müller W., Weichel W., Radbruch A. (1990). High gradient magnetic cell separation with MACS. Cytom. Part A.

[40] Hassan E.M., Willmore W.G., DeRosa M.C. (2016). Aptamers: Promising tools for the detection of circulating tumor cells. Nucl. Acid Ther..

[41] Karrer E.E., Lincoln J.E., Hogenhout S., Bennett A.B., Bostock R.M., Martineau B., Lucas W.J., Gilchrist D.G., Alexander D. (1995). In situ isolation of mRNA from individual plant cells: Creation of cell-specific cDNA libraries. Proc. Natl. Acad. Sci. USA.

[42] Rodriguez I.R., Chader G.J. (1992). A novel method for the isolation of tissue-specific genes. Nucl. Acids Res..

[43] Maher C.A., Kumar-Sinha C., Cao X., Kalyana-Sundaram S., Han B., Jing X., Sam L., Barrette T., Palanisamy N., Chinnaiyan A.M. (2009). Transcriptome sequencing to detect gene fusions in cancer. Nature.

[44] Rogers S., Macheda M.L., Docherty S.E., Carty M.D., Henderson M.A., Soeller W.C., Gibbs E.M., James D.E., Best J.D. (2002). Identification of a novel glucose transporter-like protein—GLUT-12. Am. J. Physiol. Endocrinol. Metab..

[45] Helmkampf M., Bruchhaus I., Hausdorf B. (2008). Phylogenomic analyses of lophophorates (brachiopods, phoronids and bryozoans) confirm the Lophotrochozoa concept. Proc. R. Soc. Lond. B Biol. Sci..

[46] Franzreb M., Siemann-Herzberg M., Hobley T.J., Thomas O.R. (2006). Protein purification using magnetic adsorbent particles. Appl. Microbiol. Biotechnol..

[47] Kökpinar Ö., Walter J.G., Shoham Y., Stahl F., Scheper T. (2011). Aptamer-based downstream processing of his-tagged proteins utilizing magnetic beads. Biotechnol. Bioeng..

[48] Lönne M., Bolten S., Lavrentieva A., Stahl F., Scheper T., Walter J.-G. (2015). Development of an aptamer-based affinity purification method for vascular endothelial growth factor. Biotechnol. Rep..

[49] Song F., Zhou Y., Li Y., Meng X., Meng X., Liu J., Lu S., Ren H., Hu P., Liu Z. (2014). A rapid immunomagnetic beads-based immunoassay for the detection of β-casein in bovine milk. Food Chem..

[50] Morozov V.N., Groves S., Turell M.J., Bailey C. (2007). Three minutes-long electrophoretically assisted zeptomolar microfluidic immunoassay with magnetic-beads detection. J. Am. Chem. Soc..

[51] Modh H.B., Bhadra A.K., Patel K.A., Chaudhary R.K., Jain N.K., Roy I. (2016). Specific detection of tetanus toxoid using an aptamer-based matrix. J. Biotechnol..

[52] Ilgu M., Nilsen-Hamilton M. (2016). Aptamers in analytics. Analyst.

[53] Modh H., Scheper T., Walter J.G. (2017). Detection of ochratoxin A by aptamer-assisted real-time PCR-based assay (Apta-qPCR). Eng. Life Sci..

[54] Modh H., Witt M., Urmann K., Lavrentieva A., Segal E., Scheper T., Walter J.G. (2017). Aptamer-based detection of adenosine triphosphate via qPCR. Talanta.

[55] Erdem A., Congur G. (2014). Voltammetric aptasensor combined with magnetic beads assay developed for detection of human activated protein C. Talanta.

[56] Zhao J., Lin F., Yi Y., Huang Y., Li H., Zhang Y., Yao S. (2012). Dual amplification strategy of highly sensitive thrombin amperometric aptasensor based on chitosan-Au nanocomposites. Analyst.

[57] Centi S., Tombelli S., Minunni M., Mascini M. (2007). Aptamer-based detection of plasma proteins by an electrochemical assay coupled to magnetic beads. Anal. Chem..

[58] Sun D., Lu J., Zhong Y., Yu Y., Wang Y., Zhang B., Chen Z. (2016). Sensitive electrochemical aptamer cytosensor for highly specific detection of cancer cells based on the hybrid nanoelectrocatalysts and enzyme for signal amplification. Biosens. Bioelectron..

[59] Zheng J., Zhang M., Guo X., Wang J., Xu J. (2017). Boronic acid functionalized magnetic composites with sandwich-like nanostructures as a novel matrix for PDGF detection. Sens. Actuators B Chem..

[60] Yang C., Wang Q., Xiang Y., Yuan R., Chai Y. (2014). Target-induced strand release and thionine-decorated gold nanoparticle amplification labels for sensitive electrochemical aptamer-based sensing of small molecules. Sens. Actuators B Chem..

[61] Wu D., Wang Y., Zhang Y., Ma H., Pang X., Hu L., Du B., Wei Q. (2016). Facile fabrication of an electrochemical aptasensor based on magnetic electrode by using streptavidin modified magnetic beads for sensitive and specific detection of Hg^2+^. Biosens. Bioelectron..

[62] Miao P., Yang D., Chen X., Guo Z., Tang Y. (2017). Voltammetric determination of tumor necrosis factor-α based on the use of an aptamer and magnetic nanoparticles loaded with gold nanoparticles. Microchim. Acta.

[63] Hao N., Jiang L., Qian J., Wang K. (2016). Ultrasensitive electrochemical Ochratoxin A aptasensor based on CdTe quantum dots functionalized graphene/Au nanocomposites and magnetic separation. J. Electroanal. Chem..

[64] Zelada-Guillén G.A., Tweed-Kent A., Niemann M., Göringer H.U., Riu J., Rius F.X. (2013). Ultrasensitive and real-time detection of proteins in blood using a potentiometric carbon-nanotube aptasensor. Biosens. Bioelectron..

[65] Ding J., Lei J., Ma X., Gong J., Qin W. (2014). Potentiometric aptasensing of Listeria monocytogenes using protamine as an indicator. Anal. Chem..

[66] Zhao G., Ding J., Yu H., Yin T., Qin W. (2016). Potentiometric aptasensing of *Vibrio alginolyticus* Based on DNA nanostructure—Modified magnetic beads. Sensors.

[67] Jia F., Duan N., Wu S., Dai R., Wang Z., Li X. (2016). Impedimetric salmonella aptasensor using a glassy carbon electrode modified with an electrodeposited composite consisting of reduced graphene oxide and carbon nanotubes. Microchim. Acta.

[68] Jin S., Ye Z., Wang Y., Ying Y. (2017). A novel impedimetric microfluidic analysis system for transgenic protein Cry1Ab detection. Sci. Rep..

[69] Wang Y., Ye Z., Ping J., Jing S., Ying Y. (2014). Development of an aptamer-based impedimetric bioassay using microfluidic system and magnetic separation for protein detection. Biosens. Bioelectron..

[70] Ke H., Sha H., Wang Y., Guo W., Zhang X., Wang Z., Huang C., Jia N. (2018). Electrochemiluminescence resonance energy transfer system between GNRs and Ru(bpy)_3_^2+^: Application in magnetic aptasensor for β-amyloid. Biosens. Bioelectron..

[71] Wang Y.-Z., Hao N., Feng Q.-M., Shi H.-W., Xu J.-J., Chen H.-Y. (2016). A ratiometric electrochemiluminescence detection for cancer cells using g-C_3_N_4_ nanosheets and Ag-PAMAM-luminol nanocomposites. Biosens. Bioelectron..

[72] Zhang J., Zhang X., Yang G., Chen J., Wang S. (2013). A signal-on fluorescent aptasensor based on Tb^3+^ and structure-switching aptamer for label-free detection of ochratoxin A in wheat. Biosens. Bioelectron..

[73] Fang S., Wang C., Xiang J., Cheng L., Song X., Xu L., Peng R., Liu Z. (2014). Aptamer-conjugated upconversion nanoprobes assisted by magnetic separation for effective isolation and sensitive detection of circulating tumor cells. Nano Res..

[74] Lu C., Tang Z., Liu C., Kang L., Sun F. (2015). Magnetic-nanobead-based competitive enzyme-linked aptamer assay for the analysis of oxytetracycline in food. Anal. Bioanal. Chem..

[75] Wang L., Liu F., Sui N., Liu M., William W.Y. (2016). A colorimetric assay for Hg(II) based on the use of a magnetic aptamer and a hybridization chain reaction. Microchim. Acta.

[76] Xi Z., Huang R., Li Z., He N., Wang T., Su E., Deng Y. (2015). Selection of HBsAg-specific DNA aptamers based on carboxylated magnetic nanoparticles and their application in the rapid and simple detection of hepatitis B virus infection. ACS Appl. Mater. Interfaces.

[77] Chang K.-W., Li J., Yang C.-H., Shiesh S.-C., Lee G.-B. (2015). An integrated microfluidic system for measurement of glycated hemoglobin Levels by using an aptamer-antibody assay on magnetic beads. Biosens. Bioelectron..

[78] Chen Q., Yang M., Yang X., Li H., Guo Z., Rahma M. (2018). A large Raman scattering cross-section molecular embedded SERS aptasensor for ultrasensitive Aflatoxin B1 detection using CS-Fe_3_O_4_ for signal enrichment. Spectrochim. Acta Part A Mol. Biomol. Spectrosc..

[79] Yang M., Liu G., Mehedi H.M., Ouyang Q., Chen Q. (2017). A universal sers aptasensor based on DTNB labeled GNTs/Ag core-shell nanotriangle and CS-Fe_3_O_4_ magnetic-bead trace detection of Aflatoxin B1. Anal. Chim. Acta.

[80] He J., Li G., Hu Y. (2015). Aptamer recognition induced target-bridged strategy for proteins detection based on magnetic chitosan and silver/chitosan nanoparticles using surface-enhanced Raman spectroscopy. Anal. Chem..

[81] Ozalp V.C., Bayramoglu G., Erdem Z., Arica M.Y. (2015). Pathogen detection in complex samples by quartz crystal microbalance sensor coupled to aptamer functionalized core—Shell type magnetic separation. Anal. Chim. Acta.

[82] Pan Y., Guo M., Nie Z., Huang Y., Pan C., Zeng K., Zhang Y., Yao S. (2010). Selective collection and detection of leukemia cells on a magnet-quartz crystal microbalance system using aptamer-conjugated magnetic beads. Biosens. Bioelectron..

[83] Ma C., Wang W., Yang Q., Shi C., Cao L. (2011). Cocaine detection via rolling circle amplification of short DNA strand separated by magnetic beads. Biosens. Bioelectron..

[84] Csordas A., Gerdon A.E., Adams J.D., Qian J., Oh S.S., Xiao Y., Soh H.T. (2010). Detection of proteins in serum by micromagnetic aptamer PCR (MAP) technology. Angew. Chem. Int. Ed..

[85] Ozalp V.C., Bayramoglu G., Kavruk M., Keskin B.B., Oktem H.A., Arica M.Y. (2014). Pathogen detection by core—Shell type aptamer-magnetic preconcentration coupled to real-time PCR. Anal. Biochem..

[86] Wang J. (2008). Electrochemical glucose biosensors. Chem. Rev..

[87] Han K., Liu T., Wang Y., Miao P. (2016). Electrochemical aptasensors for detection of small molecules, macromolecules, and cells. Rev. Anal. Chem..

[88] Meirinho S.G., Dias L.G., Peres A.M., Rodrigues L.R. (2016). Voltammetric aptasensors for protein disease biomarkers detection: A review. Biotechnol. Adv..

[89] Feng L., Zhang Z., Ren J., Qu X. (2014). Functionalized graphene as sensitive electrochemical label in target-dependent linkage of split aptasensor for dual detection. Biosens. Bioelectron..

[90] Yan Z., Gan N., Wang D., Cao Y., Chen M., Li T., Chen Y. (2015). A “signal-on’’aptasensor for simultaneous detection of chloramphenicol and polychlorinated biphenyls using multi-metal ions encoded nanospherical brushes as tracers. Biosens. Bioelectron..

[91] Bakker E., Pretsch E. (2008). Nanoscale potentiometry. TRAC Trends Anal. Chem..

[92] Ding J., Gu Y., Li F., Zhang H., Qin W. (2015). DNA nanostructure-based magnetic beads for potentiometric aptasensing. Anal. Chem..

[93] Lee C.-Y., Fan H.-T., Hsieh Y.-Z. (2018). Disposable aptasensor combining functional magnetic nanoparticles with rolling circle amplification for the detection of prostate-specific antigen. Sens. Actuators B Chem..

[94] Palchetti I., Mascini M. (2012). Electrochemical nanomaterial-based nucleic acid aptasensors. Anal. Bioanal. Chem..

[95] Zhou Y., Yan D., Wei M. (2015). A 2D quantum dot-based electrochemiluminescence film sensor towards reversible temperature-sensitive response and nitrite detection. J. Mater. Chem. C.

[96] Liu Z., Qi W., Xu G. (2015). Recent advances in electrochemiluminescence. Chem. Soc. Rev..

[97] Luo Z., Wang Y., Lu X., Chen J., Wei F., Huang Z., Zhou C., Duan Y. (2017). Fluorescent aptasensor for antibiotic detection using magnetic bead composites coated with gold nanoparticles and a nicking enzyme. Anal. Chim. Acta.

[98] Haase M., Schäfer H. (2011). Upconverting nanoparticles. Angew. Chem. Int. Ed..

[99] Liu Y., Tu D., Zhu H., Chen X. (2013). Lanthanide-doped luminescent nanoprobes: Controlled synthesis, optical spectroscopy, and bioapplications. Chem. Soc. Rev..

[100] Wang W., Xu D.-D., Pang D.-W., Tang H.-W. (2017). Fluorescent sensing of thrombin using a magnetic nano-platform with aptamer-target-aptamer sandwich and fluorescent silica nanoprobe. J. Lumin..

[101] Bruno J.G., Phillips T., Carrillo M.P., Crowell R. (2009). Plastic-adherent DNA aptamer-magnetic bead and quantum dot sandwich assay for Campylobacter detection. J. Fluoresc..

[102] Hao L., Zhao Q. (2016). Using fluoro modified RNA aptamers as affinity ligands on magnetic beads for sensitive thrombin detection through affinity capture and thrombin catalysis. Anal. Methods.

[103] Jin Y., Bai J., Li H. (2010). Label-free protein recognition using aptamer-based fluorescence assay. Analyst.

[104] Tan Y., Zhang X., Xie Y., Zhao R., Tan C., Jiang Y. (2012). Label-free fluorescent assays based on aptamer—Target recognition. Analyst.

[105] McKeague M., Velu R., Hill K., Bardóczy V., Mészáros T., DeRosa M.C. (2014). Selection and characterization of a novel DNA aptamer for label-free fluorescence biosensing of ochratoxin A. Toxins.

[106] Zhu Z., Yang C., Zhou X., Qin J. (2011). Label-free aptamer-based sensors for L-argininamide by using nucleic acid minor groove binding dyes. Chem. Commun..

[107] Babendure J.R., Adams S.R., Tsien R.Y. (2003). Aptamers switch on fluorescence of triphenylmethane dyes. J. Am. Chem. Soc..

[108] Huang C.-C., Chang H.-T. (2008). Aptamer-based fluorescence sensor for rapid detection of potassium ions in urine. Chem. Commun..

[109] Liang G., Cai S., Zhang P., Peng Y., Chen H., Zhang S., Kong J. (2011). Magnetic relaxation switch and colorimetric detection of thrombin using aptamer-functionalized gold-coated iron oxide nanoparticles. Anal. Chim. Acta.

[110] Kim Y.S., Jurng J. (2013). A simple colorimetric assay for the detection of metal ions based on the peroxidase-like activity of magnetic nanoparticles. Sens. Actuators B Chem..

[111] Wang C., Qian J., Wang K., Yang X., Liu Q., Hao N., Wang C., Dong X., Huang X. (2016). Colorimetric aptasensing of ochratoxin A using Au@Fe_3_O_4_ nanoparticles as signal indicator and magnetic separator. Biosens. Bioelectron..

[112] Zhang Z., Wang Z., Wang X., Yang X. (2010). Magnetic nanoparticle-linked colorimetric aptasensor for the detection of thrombin. Sens. Actuators B Chem..

[113] Yan X., Cao Z., Kai M., Lu J. (2009). Label-free aptamer-based chemiluminescence detection of adenosine. Talanta.

[114] Li Y., Ji X., Liu B. (2011). Chemiluminescence aptasensor for cocaine based on double-functionalized gold nanoprobes and functionalized magnetic microbeads. Anal. Bioanal. Chem..

[115] Sun C., Zhang R., Gao M., Zhang X. (2015). A rapid and simple method for efficient capture and accurate discrimination of circulating tumor cells using aptamer conjugated magnetic beads and surface-enhanced Raman scattering imaging. Anal. Bioanal. Chem..

[116] Yoon J., Choi N., Ko J., Kim K., Lee S., Choo J. (2013). Highly sensitive detection of thrombin using SERS-based magnetic aptasensors. Biosens. Bioelectron..

[117] Teller C., Halámek J., Makower A., Scheller F.W. (2009). A set of piezoelectric biosensors using cholinesterases. Biosensors and Biodetection.

[118] Skládal P. (2016). Piezoelectric biosensors. TRAC Trends Anal. Chem..

[119] Song W., Zhu Z., Mao Y., Zhang S. (2014). A sensitive quartz crystal microbalance assay of adenosine triphosphate via DNAzyme-activated and aptamer-based target-triggering circular amplification. Biosens. Bioelectron..

[120] Feng J., Dai Z., Tian X., Jiang X. (2018). Detection of Listeria monocytogenes based on combined aptamers magnetic capture and loop-mediated isothermal amplification. Food Control.

